# Identification of Calcium Sulphoaluminate Formation between Alunite and Limestone

**DOI:** 10.3390/s90705059

**Published:** 2009-06-25

**Authors:** Hyung-Seok Kim, Gi-Chun Han, Ji-Whan Ahn, Kye-Hong Cho, Hee-Chan Cho

**Affiliations:** 1 Mineral Resources Research Division, Korea Institute of Geoscience & Mineral Resources, 30 Gajeong-dong, Yuseong-gu, Daejeon 305-350, Korea; E-Mails: hskim@kigam.re.kr (H.K.); ahnjw@kigam.re.kr (J.A.); khcho99@paran.com (K.C.); 2 Department of Energy Resources Engineering, Seoul National University, 599 Gwanak-ro, Gwanak-gu, Seoul 151-742, Korea; E-Mail: hccho@snu.ac.kr (H.C.)

**Keywords:** alunite, limestone, calcium sulphoaluminate, calcium langbeinite

## Abstract

This study was carried out to identify the conditions of formation of calcium sulphoaluminate (3CaO·3Al_2_O_3_·CaSO_4_) by the sintering of a limestone (CaCO_3_) and alunite [K_2_SO_4_·Al_2_(SO_4_)_3_·4Al(OH)_3_] mixture with the following reagents: K_2_SO_4_, CaCO_3_, Al(OH)_3_, CaSO_4_·2H_2_O, and SiO_2_. When K_2_SO_4_, CaCO_3_, Al(OH)_3_, CaSO_4_·2H_2_O were mixed in molar ratios of 1:3:6:3 and sintered at 1,200∼1,300 °C, only 3CaO·3Al_2_O_3_·CaSO_4_ and calcium langbeinite (2CaSO_4_·K_2_SO_4_) were generated. With an amount of CaO that is less than the stoichiometric molar ratio, 3CaO·3Al_2_O_3_·CaSO_4_ was formed and anhydrite (CaSO_4_) did not react and remained behind. With the amount of CaSO_4_ that is less than the stoichiometric molar ratio, the amounts of 3CaO·3Al_2_O_3_·CaSO_4_ and 2CaSO_4_·K_2_SO_4_ decreased, and that of CaO·Al_2_O_3_ increased. In the K_2_SO_4_-CaO-Al_2_O_3_-CaSO_4_-SiO_2_ system, to stabilize the formation of 3CaO·3Al_2_O_3_·CaSO_4_, 2CaSO_4_·K_2_SO_4_, and β-2CaO·SiO_2_, the molar ratios of CaO: Al_2_O_3_: CaSO_4_ must be kept at 3:3:1 and that of CaO/SiO_2_, over 2.0; otherwise, the generated amount of 3CaO·3Al_2_O_3_·CaSO_4_ decreased and that of gehlenite (2CaO·Al_2_O_3_·SiO_2_) with no hydration increased quantitatively. Therefore, if all SO_3_(g) generated by the thermal decomposition of alunite reacts with CaCO_3_ (or CaO, the thermal decomposition product of limestone) to form CaSO_4_ in an alunite- limestone system, 1 mol of pure alunite reacts with 6 mol of limestone to form 1 mol of 3CaO·3Al_2_O_3_·CaSO_4_ and 1 mol of 2CaSO_4_·K_2_SO_4_.

## 1. Introduction

Cement has been prepared using alunite [1], and a type of special cement containing calcium aluminate, anhydrite, and potassium sulfate has been prepared using anhydrite formed by the reaction between SO_3_(g) that evolves from alunite and limestone via the following reaction:

$$K_{2}{\text{SO}}_{4}\cdot {\text{Al}}_{2}{\left({\text{SO}}_{4}\right)}_{3}\cdot 4\text{Al}{\left(\text{OH}\right)}_{3}+{\text{mCaCO}}_{3}\to K_{2}{\text{SO}}_{4}+3(\text{nCaO}\cdot {\text{Al}}_{2}O_{3})+3{\text{CaSO}}_{4}+{\text{mCO}}_{2}(g)+6H_{2}O\;(g)$$

Choi *et al.* [2] synthesized a calcium sulphoaluminate clinker consisting of 3CaO·3Al_2_O_3_·CaSO_4_, CaO and CaSO_4_ through the sintering for 2 hrs at a temperature of 1,200 °C of alunite, limestone and an anhydrite mixture at a weight ratio of 1:13:5. Their research results showed that alunite could be used in the preparation of a 3CaO·3Al_2_O_3_·CaSO_4_ clinker provided that adequate mixing conditions are provided, despite that the expansion is relatively small compared to that of a clinker synthesized from reagents. However, the exact formation conditions of 3CaO·3Al_2_O_3_·CaSO_4_ in the sintering state were not discussed. In addition, Han *et al.* [3,4] synthesized a clinker containing calcium fluoroaluminate (C_11_A_7_·CaF_2_) from domestic alunite and investigated its characteristics in an effort to develop a type of fast-hardening cement.

The authors [5] carried out an investigation of the conditions under which 3CaO·3Al_2_O_3_·CaSO_4_ is formed when mixtures of alunite and limestone are sintered. It was concluded that calcium langbeinite (2CaSO_4_·K_2_SO_4_) forms from 700 °C and that calcium sulphoaluminate forms from 800 °C. Both are stable up to 1,300 °C, as shown in the equation below:

$$K_{2}{\text{SO}}_{4}\cdot {\text{Al}}_{2}{\left({\text{SO}}_{4}\right)}_{3}\cdot 4\text{Al}{\left(\text{OH}\right)}_{3}+6{\text{CaCO}}_{3}\to 4\text{CaO}\cdot 3{\text{Al}}_{2}O_{3}\cdot {\text{SO}}_{3}+2{\text{CaSO}}_{4}\cdot K_{2}{\text{SO}}_{4}+{6H}_{2}O(g)+{6\text{CO}}_{2}(g)$$

However, when alunite or limestone is incorporated in SiO_2_ so as to enable the formation of calcium sulphoaluminate, the molar ratios of CaO/alunite and CaO/SiO_2_ must be kept over 6.0 and 2.0, respectively. A clinker composed of calcium sulphoaluminate and calcium langbeinite was transformed into ettringite(3CaO·Al_2_O_3_·3CaSO_4_·32H_2_O) in water as calcium langbeinite is transformed into CaSO_4_·2H_2_O(s) and K_2_SO_4_ (aq) in water.

In the present study, the formation of 3CaO·3Al_2_O_3_·CaSO_4_ is identified in a K_2_SO_4_-CaO-Al_2_O_3_-CaSO_4_-SiO_2_ system using reagents of various types in a detailed investigation of the formation conditions of this species through the sintering of a mixture of alunite and limestone.

## 2. Experimental

The reagents listed in Table 1 were used to investigate the reaction products arising from the K_2_SO_4_-CaO-Al_2_O_3_-CaSO_4_-SiO_2_ system. K_2_SO_4_ and Al(OH)_3_ were used in substitution for the components of alunite; calcium carbonate (CaCO_3_) substituted for limestone, and CaSO_4_·2H_2_O, substituted for anhydrite (CaSO_4_), which is formed by the sintering reaction between alunite and limestone.

The mixture of reagents were sintered in a programmable electric furnace (Barnstead /Thermolyne F46120 CM High-Temperature Furnace : 240 V, 40 A, 2,500 Watt, 50/60 Hz, 1 Phase) below 1,300 °C in an air atmosphere. The mineral phases of the manufactured clinker were then analyzed by XRD(PW-1700, Philips, 30kV, 25mA, Cu target, Ni filter, at a scanning rate of 2^°^/min). After the prepared clinker was finely milled with a laboratory ball mill, the mineral phases of the crushed clinker were analyzed by XRD.

## 3. Results and Discussion

Alunite (K_2_SO_4_·Al_2_(SO_4_)_3_·4Al(OH)) is transformed into KAl(SO_4_)_2_ and Al_2_O_3_ by dehydration at 500∼580 °C and KAl(SO_4_)_2_, K_2_SO_4_ and Al_2_O_3_ by desulphurization at 700∼780 °C via the reaction of K_2_SO_4_·Al_2_(SO_4_)_3_·4Al(OH)_3_ → K_2_SO_4_ + 2Al_2_O_3_ + 3SO_3_(g) + 6H_2_O(g), regardless of the partial pressure of CO_2_(g). However, limestone decomposes from 650 °C in air and from 900 °C in a CO_2_(g) saturated atmosphere [6].

When the mixture of alunite and limestone is sintered in air and in a CO_2_(g) saturated atmosphere, the rate of formation of anhydrite is relatively low, at 76.0% and 67.0%, respectively, at a CaCO_3_/alunite stoichiometric molar ratio of 3. However, the rate of formation increases as the molar ratio of CaCO_3_/alunite (particle size, 37∼44 μm) exceeds 6, showing rates of more than 99.0% and 95.0% in air and in a CO_2_(g) saturated atmosphere, respectively [7].

As shown in the results of aforementioned experiment, if alunite and limestone are mixed and sintered in air, most of the generated SO_3_ reacts with limestone to form anhydrite (CaSO_4_). Additionally, ignoring impurities such as Fe_2_O_3_, TiO_2_, and P_2_O_5_ included in the alunite, because alunite ore is composed of alunite, quartz(SiO_2_), and the aluminum silicate minerals of kaolinite, dickite, and pyrophyllite, the alunite and limestone mixture can be said to have five components, as does K_2_SO_4_-CaO-Al_2_O_3_-CaSO_4_-SiO_2_.

If 1 mol if pure alunite is heated to temperatures that exceed 800 °C, it is pyrolyzed as K_2_SO_4_·Al_2_(SO_4_)_3_·4Al(OH)_3_ → K_2_SO_4_ + 3Al_2_O_3_ + 3SO_3_ + 6H_2_O. Therefore, to formulate SO_3_(g) of 3 mol into anhydrite, 3 mol of CaCO_3_ is required, and to change 3 mol of Al_2_O_3_ into 3CaO·3Al_2_O_3_·CaSO_4_, 3 additional mol of CaCO_3_ and 1 mol of CaSO_4_ will be needed. Therefore, to change all of the Al_2_O_3_ in alunite into 3CaO·3Al_2_O_3_·CaSO_4_, and to generate SO_3_(g) by the thermal decomposition of alunite into anhydrite, theoretically, pure alunite and limestone should be mixed at a molar rate of 1:6. This mixture will be composed of five component systems as in K_2_SO_4_-CaO-Al_2_O_3_-CaSO_4_.

The present study aims to determine the mineral phases of a clinker generated in the K_2_SO_4_-CaO-Al_2_O_3_-CaSO_4_ and the K_2_SO_4_-CaO-Al_2_O_3_-CaSO_4_-SiO_2_ systems, using several types of reagents, including K_2_SO_4_, Al(OH)_3_, SiO_2_, CaSO_4_·2H_2_O, and CaCO_3_.

**Figure 1.**
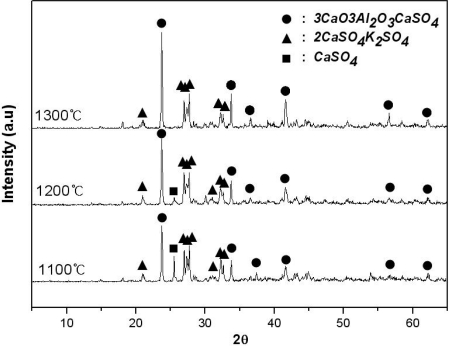
X-ray diffraction patterns of sintered products of mixtures of K_2_SO_4_-3CaO-3Al_2_O_3_-3CaSO_4_ at various temperatures in air.

Figure 1 shows X-ray diffraction patterns of the materials generated, when a compound consisting of K_2_SO_4_-3CaO-3Al_2_O_3_-3CaSO_4_ was sintered at 1,100 °C, 1,200 °C, and 1,300 °C, respectively, for 2 hours. As shown in this figure, 3CaO·3Al_2_O_3_·CaSO_4_ and 2CaSO_4_·K_2_SO_4_ is formed, but CaSO_4_ does not react and remains at 1,100 °C. Meanwhile, at temperatures in excess of 1,200 °C 3CaO·3Al_2_O_3_·CaSO_4_ and 2CaSO_4_·K_2_SO_4_ were mainly generated. Accordingly, 1 mol of pure alunite reacts with 6 mol of limestone to form 1 mol of 3CaO·3Al_2_O_3_·CaSO_4_ and 1 mol of 2CaSO_4_·K_2_SO_4_ as follows; K_2_SO_4_·Al_2_(SO_4_)_3_·4Al(OH)_3_ + 6CaCO_3_ → 2CaSO_4_·K_2_SO_4_ + 3CaO·3Al_2_O_3_·CaSO_4_ + 6H_2_O(g) + 6CO_2_(g).

**Figure 2.**
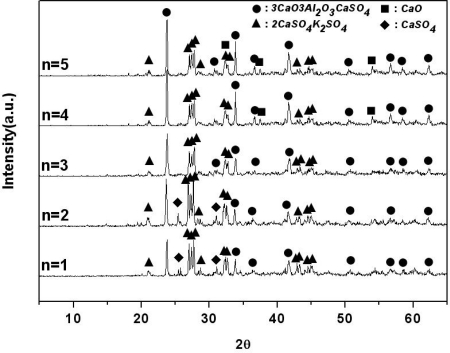
X-ray diffraction patterns of sintered products of mixtures of K_2_SO_4_-nCaO-3Al_2_O_3_-3CaSO_4_ with various amounts of CaO in air (sintering temp.: 1,200 °C, sintering time: 2 hrs).

Figure 2 displays the materials generated from K_2_SO_4_-nCaO-3Al_2_O_3_-3CaSO_4_ with various amounts of CaO sintered at 1,200 °C for two hours, as determined via an XRD analysis.

When it CaO is included in at an amount of 3 mol, 3CaO·3Al_2_O_3_·CaSO_4_ and 2CaSO_4_·K_2_SO_4_ are mainly generated. However, it was found that as the amount of CaO is increased from 3 mol to 5 mol, CaO does not participate in the formation reaction of 3CaO·3Al_2_O_3_·CaSO_4_. Thus, it can be said that 3CaO·3Al_2_O_3_·CaSO_4_ and 2CaSO_4_·K_2_SO_4_ are generated stably when CaO is 3 mol in the K_2_SO_4_-nCaO-3Al_2_O_3_-3CaSO_4_ system.

According to Fukuda [8] 3CaO·3Al_2_O_3_·CaSO_4_ is the only compound in the CaO-Al_2_O_3_-SO_3_ system. This line of research was started in 1962 by Halstead, *et al.* [9] with Ca^2+^ and SO_4_^2−^ ions in a three-dimensional crystal structure sharing the angular point of a AlO_4_ tetrahedron; an Al^3+^ ion is coordinated with four O^2−^ ions, a Ca^2+^ ion is surrounded asymmetrically by O^2−^ ions, and an isolated SO_4_^2−^ ion is characterized by its ability to readily react with water. Kondo [10] discovered that these ions become hardened in water. In general, they are produced by re-sintering after producing 3CaO·Al_2_O_3_ and regulating the mixture ratio of 3CaO·Al_2_O_3_, CaSO_4_·2H_2_O and Ca(OH)_2_ and by sintering a mixture of CaCO_3_, Al_2_O_3_, and CaSO_4_·2H_2_O.

2CaSO_4_·K_2_SO_4_ is easily generated in the CaO-Al_2_O_3_-SiO_2_-Fe_2_O_3_-MgO-CaSO_4_-K_2_SO_4_ system and has a considerable influence on the condensation time and the hardening characteristics of cements. Known as a water-soluble alkali, as 2CaSO_4_·K_2_SO_4_ can be easily separated from the liquid state of a clinker oxide and is said to become K_2_SO_4_·CaSO_4_·H_2_O(syngenite) and CaSO_4_·2H_2_O upon exposure to water [11].

**Figure 3.**
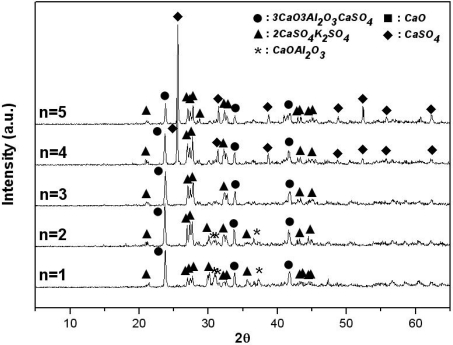
X-ray diffraction patterns of sintered products of mixtures of K_2_SO_4_-3CaO-3Al_2_O_3_-nCaSO_4_ with various amounts of CaSO_4_ in air (sintering temp.: 1,200 °C, sintering time: 2 hrs).

Figure 3 shows the materials generated from the K_2_SO_4_-3CaO-3Al_2_O_3_-nCaSO_4_ system sintered at 1,200 °C for two hours with varying amounts of anhydrite, as determined via an XRD analysis.

As shown in this figure, when anhydrite is included in an amount of 3 mol, 3CaO·3Al_2_O_3_·CaSO_4_ and 2CaSO_4_·K_2_SO_4_ are generated. However, as the amount of anhydrite is decreased from 3 mol to 1 mol, it was observed that the K_2_SO_4_ that did not react with CaO·Al_2_O_3_ remained. As the amount of anhydrite is increased from 3 mol to 5 mol, only the diffraction strength of the CaSO_4_ that does not react increases. Thus, it can be said that an amount of CaSO_4_ in excess of the stoichiometric molar ratio is necessary to generate 3CaO·3Al_2_O_3_·CaSO_4_ stably.

As noted above, 3CaO·3Al_2_O_3_·CaSO_4_ and 2CaSO_4_·K_2_SO_4_ were mainly generated in the K_2_SO_4_-3CaO-3Al_2_O_3_-3CaSO_4_ system. However, because alunite from nature has impurities of SiO_2_ and aluminum silicate minerals such as kaolinite, dickite, and pyrophyllite, the alunite and limestone mixture is believed to be comprised of a K_2_SO_4_-CaO-Al_2_O_3_-CaSO_4_-SiO_2_ system to which SiO_2_ is added.

**Figure 4.**
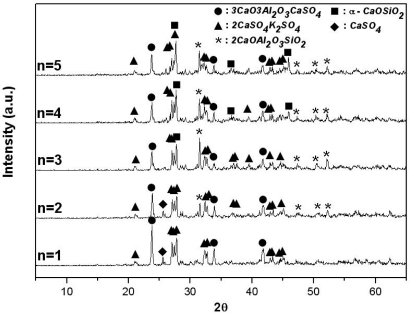
X-ray diffraction patterns of sintered products of mixtures of K_2_SO_4_-3CaO-3Al_2_O_3_-3CaSO_4_-nSiO_2_ with various amounts of SiO_2_ in air (sintering temp.: 1,200 °C, sintering time: 2 hrs).

**Figure 5.**
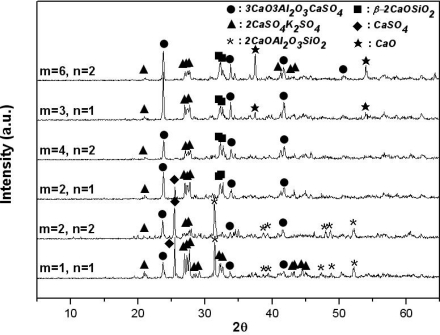
X-ray diffraction patterns of sintered products of mixtures of K_2_SO_4_-[(3+m)CaO]-3Al_2_O_3_-3CaSO_4_-nSiO_2_ with various amounts of CaO and SiO_2_ in air (sintering temp.: 1,200 °C, sintering time: 2 hrs).

Figures 4 and 5 show, by XRD analysis, the materials generated from the K_2_SO_4_-3CaO-3Al_2_O_3_-3CaSO_4_-nSiO_2_ system and the K_2_SO_4_-(3+m)CaO-3Al_2_O_3_-3CaSO_4_-nSiO_2_ system, respectively, sintered at 1,200 °C for 2 hours, with various amounts of CaO (3 + m : mol number) and SiO_2_(n: mol number). In Figure 4, when there is no SiO_2_ in the mixture, the result is identical to that obtained when 3 mol of CaO is used (Figure 2). It is expected that 3CaO·3Al_2_O_3_·CaSO_4_ and 2CaSO_4_·K_2_SO_4_ will be generated. However, as the amount of SiO_2_ is increased from 1 mol to 5 mol, the amount of 3CaO·3Al_2_O_3_·CaSO_4_ generated is reduced, because gehlenite (2CaO·Al_2_O_3_·SiO_2_) and wollastonite (α-CaO·SiO_2_), which do not react with water, are generated. In addition, as shown in Figure 5, when the mol rate of CaO/alunite is less than 6 (m less than 3), and that of CaO/SiO_2_ is less than 2, the synthetic clinker contained 2CaO·Al_2_O_3_·SiO_2_ and CaSO_4_; these compounds do not participate in the formation reaction of 3CaO·3Al_2_O_3_·CaSO_4_ because 3CaO·3Al_2_O_3_·CaSO_4_ is the only compound in the CaO-Al_2_O_3_-SO_3_ system as noted above[8] and CaO and Al_2_O_3_ participate preferentially in the formation reaction of 2CaO·Al_2_O_3_·SiO_2_. Accordingly, if all SO_3_(g) generated by thermal decomposition of alunite reacts with CaCO_3_ (or CaO, the thermal decomposition product of limestone) to form CaSO_4_ in the alunite-limestone system, when the molar ratios of CaO/alunite exceed 6 and that of CaO/SiO_2_ exceeds 2, 3CaO·3Al_2_O_3_·CaSO_4_, 2CaSO_4_·K_2_SO_4_ and β-2CaO·SiO_2_ are generated stably.

## 4. Conclusions

This study investigated the formation characteristics of calcium sulphoaluminate (4CaO·3Al_2_O_3_·SO_3_) in a K_2_SO_4_-CaO-Al_2_O_3_-CaSO_4_-SiO_2_ system using reagents of various types in an effort to identify the formation conditions of 3CaO·3Al_2_O_3_·CaSO_4_ by the sintering of a mixture of alunite and limestone. In experiments with reagents for the mineral phases generated from the compound system (the K_2_SO_4_-CaO-Al_2_O_3_-CaSO_4_-SiO_2_ system), if all SO_3_(g) from alunite reacts with limestone to form anhydrite, it was found that 1 mol of pure alunite reacts with 6mol of limestone to form 1 mol of 3CaO·3Al_2_O_3_·CaSO_4_ and 1 mol of 2CaSO_4_·K_2_SO_4_, as of 3CaO·3Al_2_O_3_·CaSO_4_ and 2CaSO_4_·K_2_SO_4_ are generated stably at a component ratio of K_2_SO_4_-3CaO-3Al_2_O_3_-3CaSO_4_. Over-mixing of CaO and CaSO_4_ has a slight effect on the generation of 3CaO·3Al_2_O_3_·CaSO_4_. However, it is thought that under-mixing will have a substantial effect on the mineral phase of the clinker and on the amount of 3CaO·3Al_2_O_3_·CaSO_4_ that is generated. Moreover, if the impurities of SiO_2_ are incorporated in the alunite and limestone, the molar ratio of CaO/alunite must exceed 6 and that of CaO/SiO_2_ must exceed 2 in order to ensure the stable formation of calcium sulphoaluminate, calcium langbeinite, and the β-2CaO·SiO_2_ phases. Otherwise, the amount of gehlenite(2CaO·Al_2_O_3_·SiO_2_), which does not react with water, may be increased.

## Figures and Tables

**Table 1.** List of reagents used in this study.

**Reagents**	**Chemical formula**	**Purity**	**Manufacturer**
Potassium Sulfate	K_2_SO_4_	First grade	Ducksan Pharmaceutical Co., Ltd
Aluminum Hydroxide	Al(OH)_3_	First grade	Shinyo Pure Chemical Co., Ltd
Calcium Carbonate	CaCO_3_	min 98.0%	Kanto Chemical Co., Inc.
Gypsum	CaSO_4_·2H_2_O	Extra pure	Junsei Chemical Co., Ltd.
Silicate Dioxide	SiO_2_	Extra pure	Junsei Chemical Co., Ltd.
